# Deciphering the Mechanisms of Improved Immunogenicity of Hypochlorous Acid-Treated Antigens in Anti-Cancer Dendritic Cell-Based Vaccines

**DOI:** 10.3390/vaccines8020271

**Published:** 2020-06-02

**Authors:** Michele Graciotti, Fabio Marino, HuiSong Pak, Petra Baumgaertner, Anne-Christine Thierry, Johanna Chiffelle, Marta A. S. Perez, Vincent Zoete, Alexandre Harari, Michal Bassani-Sternberg, Lana E. Kandalaft

**Affiliations:** 1 Department of Oncology, Ludwig Institute for Cancer Research, University of Lausanne, 1011 Lausanne, Switzerland; michele.graciotti@chuv.ch (M.G.); fabio.marino@ervaxx.com (F.M.); Hui-Song.Pak@chuv.ch (H.P.); Petra.Baumgartner@hospvd.ch (P.B.); Anne-Christine.Thierry@chuv.ch (A.-C.T.); Johanna.Chiffelle@chuv.ch (J.C.); marta.perez@unil.ch (M.A.S.P.); vincent.zoete@unil.ch (V.Z.); Alexandre.Harari@chuv.ch (A.H.); 2 Center of Experimental Therapeutics, Department of Oncology, University Hospital of Lausanne (CHUV), 1011 Lausanne, Switzerland; 3 Swiss Institute of Bioinformatics, 1015 Lausanne, Switzerland

**Keywords:** dendritic cells, cancer vaccines, immunotherapy, proteomics, immunopeptidomics

## Abstract

Hypochlorous acid (HOCl)-treated whole tumor cell lysates (Ox-L) have been shown to be more immunogenic when used as an antigen source for therapeutic dendritic cell (DC)-based vaccines, improving downstream immune responses both in vitro and in vivo. However, the mechanisms behind the improved immunogenicity are still elusive. To address this question, we conducted a proteomic and immunopeptidomics analyses to map modifications and alterations introduced by HOCl treatment using a human melanoma cell line as a model system. First, we show that one-hour HOCl incubation readily induces extensive protein oxidation, mitochondrial biogenesis, and increased expression of chaperones and antioxidant proteins, all features indicative of an activation of oxidative stress-response pathways. Characterization of the DC proteome after loading with HOCl treated tumor lysate (Ox-L) showed no significant difference compared to loading with untreated whole tumor lysate (FT-L). On the other hand, detailed immunopeptidomic analyses on monocyte-derived DCs (mo-DCs) revealed a great increase in human leukocyte antigen class II (HLA-II) presentation in mo-DCs loaded with Ox-L compared to the FT-L control. Further, 2026 HLA-II ligands uniquely presented on Ox-L-loaded mo-DCs were identified. In comparison, identities and intensities of HLA class I (HLA-I) ligands were overall comparable. We found that HLA-II ligands uniquely presented by DCs loaded with Ox-L were more solvent exposed in the structures of their source proteins, contrary to what has been hypothesized so far. Analyses from a phase I clinical trial showed that vaccinating patients using autologous Ox-L as an antigen source efficiently induces polyfunctional vaccine-specific CD4+ T cell responses. Hence, these results suggest that the increased immunogenicity of Ox-L is, at least in part, due to qualitative and quantitative changes in the HLA-II ligandome, potentially leading to an increased HLA-II dependent stimulation of the T cell compartment (i.e., CD4+ T cell responses). These results further contribute to the development of more effective and immunogenic DC-based vaccines and to the molecular understanding of the mechanism behind HOCl adjuvant properties.

## 1. Introduction

Dendritic cells (DCs) are professional antigen-presenting cells (APCs) that play a crucial role at the interface between innate and adaptive immune system [1]. Their main function is to uptake and process foreign antigens and present them to T cell compartments, thus activating a subsequent immune response against pathogens. For this reason, DCs have been largely investigated in in vitro studies and clinical trials in adjuvant settings as an anti-cancer therapy [2]. The common therapeutic approach relies first on isolating monocyte precursors from patients’ peripheral blood, followed by ex vivo culture in the presence of granulocyte-macrophage colony-stimulating factor (GM-CSF) and interleukin-4 (IL-4) for cell differentiation into immature monocyte-derived DCs (mo-DCs). These cells are then loaded with tumor antigens (commonly peptides, proteins, or whole tumor lysates (WTLs)) and subsequently incubated with diverse stimuli (e.g., Toll-like receptors (TLRs) agonists, prostaglandins, interferon- (IFN-)) to induce cell maturation and terminal differentiation into fully competent APCs. In particular, this two-step process is essential to ensure: (i) a tumor antigen-specific immune response, (ii) the upregulation of surface markers essential for T cell stimulation (notably CD83, CD86, and HLA-I/–II molecules, among others) [3,4], and (iii) cell migration and lymphoid trafficking (e.g., through upregulation of CCR7) [5]. 

Despite its discreet success, the immunogenicity and therapeutic outcomes of DC immunotherapy are still considered suboptimal and different strategies are currently being developed to address this point [6,7]. Among these, several independent studies have demonstrated a great improvement in immune responses when, prior to DC loading, tumor antigen(s) were treated with hypochlorous acid (HOCl). HOCl is a potent oxidant with antibacterial properties, which is produced by activated neutrophils during acute inflammation [8] and is thus physiologically present at the local infection site at the onset of a mounting immune response. Early in vitro studies showed that, upon T cell incubation with APCs stimulated with HOCl-treated model antigens, such as ovalbumin (Ova) or bovine serum albumin (BSA), increased T cell responses were observed, including antigen recognition [9], cell proliferation [10], and IL-2 production [11]. Subsequent studies also demonstrated that HOCl treatment further improves antigen uptake by DCs [12,13], proteolytic digestion [9,13], and humoral responses against model proteins [14] compared to their native counterparts. Interestingly, similar observations were also made in the context of DC stimulation with HOCl-conditioned tumor cells [12,15] or WTLs [16] prior to DC antigen stimulation. In particular, these studies confirmed that HOCl antigen oxidation resulted in in vitro enhanced uptake rates by both human [16] and mouse [12] DCs, stronger T cell priming and activation, and even improved therapeutic outcomes both in a mouse model of ovarian cancer [12] and in ovarian cancer patients [16]. Importantly, follow-up analyses on these cancer patients further demonstrated significantly prolonged survival upon vaccination with DCs stimulated with HOCl-treated autologous tumor cells, as well as the emergence of de novo anti-tumor T cell responses [17].

Despite these encouraging results, the underlying cellular or molecular mechanisms behind the observed improved immunogenicity induced by HOCl, have not been identified yet. Interestingly, recent work by Biedron demonstrated that HOCl adjuvant effects are not uniquely related to improved antigen uptake, and that instead other additional mechanisms are also involved [18]. However, at present, these findings remain mostly speculative. In order to address this issue, we adopted proteomic and immunopeptidomic approaches to map and characterize the effects of HOCl oxidation at both the antigen level, using the A375 melanoma cell line as a model system, and at the DC level, using mo-DCs from healthy donors. This analysis was complemented with a bioinformatic analysis aimed at identifying differences between the pool of antigens presented by mo-DCs loaded with HOCl-treated tumor lysate or with the untreated control counterpart and the validation of its increased immunogenicity in a human clinical trial. Our results elucidate mechanisms of improved immunogenicity associated with Ox-L.

## 2. Materials and Methods

### 2.1. Whole Tumor Lysate (WTL) Preparation

WTLs were prepared as previously described [16]. Briefly, A375 tumor cells (ATCC) grown in RPMI media 1640 (Gibco, Waltham, MA, USA) supplemented with 10% fetal bovine serum (Gibco, Waltham, MA, USA) were harvested, washed, resuspended in PBS at 1 × 10^6^ cells/mL, and incubated in the presence (Ox-L) or absence (FT-L) of HOCl (Sigma-Aldrich, Buchs, Switzerland) 60 M for 1 h at 37 °C. Cells were then washed twice with PBS to ensure extensive removal of residual HOCl, resuspended in RPMI at 1 × 107 cells/mL and subjected to 6 freeze/thaw cycles. WTLs were then stored at −80 °C prior to use.

### 2.2. DC Preparation and WTL Loading

DC generation was carried out as previously described [19,20]. Briefly, monocytes were isolated from fresh peripheral blood monononuclear cells from healthy donors using CD14 microbeads (Miltenyi Biotech, Bergisch Gladbach, Germany) following manufacturer instructions and cultured at 1 × 10^6^ cells/mL in DC Medium (CellGenix, Freiburg, Germany) in the presence of 2 mM glutamine (ThermoFisher Scientific, Waltham, USA), 2% human serum (Bioconcept, Allschwil, Switzerland), 250 IU/mL IL-4 (Miltenyi Biotech, Bergisch Gladbach, Germany), and 500 IU/mL GM-CSF (Miltenyi Biotech, Bergisch Gladbach, Germany). After 4 days, cells were incubated at 37 °C with WTL at a cell ratio of 1:1 or DC medium (unloaded control) for 24 h, followed by cell maturation with 60EU/mL liposaccharide (Sigma-Aldrich, Buchs, Switzerland) and 2000 IU/mL IFN- (Miltenyi Biotech, Bergisch Gladbach, Germany) for 16 h.

### 2.3. HLA Typing

HLA typing was performed with the Trusight HLA V2 panel kit (Illumina, San Diego, CA, USA). Class I and Class II genes were amplified by PCR and Illumina adapters were added by tagmentation. After normalization and purification, the samples were sequenced on the MiSeq instrument (Illumina, San Diego, CA, USA). Sequences were aligned against the IMGT Page 1/5 database and analyzed with the Assign V2 software (Illumina, San Diego, CA, USA), and the results are available in Table S1.

### 2.4. Sample Preparation for Proteomics Analysis

Cell pellets or tumor lysates obtained from 10^6^ cells were re-suspended in lysis buffer composed of 8M Urea (Biochemica, Billingham, UK) and 50 mM ammonium bicarbonate (AMBIC, Sigma-Aldrich, Buchs, Switzerland) pH 8. The cell lysates were sonicated in the Bioruptor instrument (Diagenode, B01020001, Seraing, Belgium) for 15 cycles, maximum mA for 30 s per cycle. Subsequently, centrifugation at 20,000× *g* at 4 °C for 30 min separated the soluble from the insoluble protein fractions. The soluble fraction was collected and the protein concentration of the lysates was determined by Nanodrop. Proteins (20 μg) were reduced with a final concentration of 5 mM DTT (Sigma-Aldrich, Buchs, Switzerland) at 37 °C for 60 min, followed by alkylation with a final concentration of 15 mM iodacetamide (Sigma-Aldrich, Buchs, Switzerland) at room temperature for 60 min in the dark. After alkylation, the digestion was carried out with a mixture of endoproteinase Lys-C and Trypsin (Trypsin/Lys-c Mix, Promega, Madison, WI, USA). The first step consists of endoproteinase Lys-C digestion for 4 h at 37 °C with a protein to enzyme ratio of 50:1 (*w*/*w*). Subsequently, the samples were diluted 8 times with 50 mM AMBIC to a Urea concentration of 1M. The second step of digestion was performed with Trypsin overnight at 37 °C with a substrate to enzyme ratio of 50:1 (*w*/*w*). After digestion, the samples were acidified with formic acid (FA) and desalted on C-18 spin columns (Harvard Apparatus, Holliston, MA, USA). Finally, the samples were dried and resuspended in 2% ACN in 0.1 % FA (Thermo Fisher Scientific, Waltham, MA, USA) at a 0.6 μg/μL concentration.

### 2.5. Immunoaffinity Purification of HLA Peptides

We performed HLA immunoaffinity purification according to our previously established protocols [21,22]. Briefly, W6/32 and HB145 monoclonal antibodies were purified from the supernatants of HB95 (ATCC^®^ HB-95™) and HB145 cells (ATCC^®^ HB-145™) using protein-A sepharose 4B (Pro-A) beads (Invitrogen, Waltham, MA, USA), and antibodies were cross-linked to Pro-A beads. Cells were lysed with PBS containing 0.25% sodium deoxycholate (Sigma Aldrich, Buchs, Switzerland), 0.2 mM iodoacetamide (Sigma Aldrich, Buchs, Switzerland), 1 mM EDTA, a 1:200 protease inhibitors cocktail (Sigma Aldrich, Buchs, Switzerland), 1 mM phenylmethylsulfonylfluoride (Roche, Basel, Switzerland), and 1% octyl-beta-D glucopyranoside (Sigma-Aldrich, Buchs, Switzerland) at 4 °C for 1 h. The lysates were then cleared by centrifugation at 25,000 rpm in a high-speed centrifuge (Beckman Coulter, Nyon, Switzerland; JSS15314) at 4 °C for 50 min. We employed the Waters Positive Pressure-96 Processor (Waters, Baden-Dättwil, Switzerland) and 96-well single-use micro-plates with 3 µm glass fibers and 10 µm polypropylene membranes (Seahorse Bioscience, Lexington, KY, USA; ref no: 360063). The lysates were passed sequentially through the first plate containing W6/32 -cross-linked beads, then through the second plate with HB145-cross-linked beads, at 4 °C. The beads in the plates were then washed separately with varying concentrations of salts using the processor. Finally, the beads were washed twice with 2 mL of 20 mM Tris-HCl pH 8.

Sep-Pak tC18 100 mg Sorbent 96-well plates (Waters, Baden-Dättwil, Switzerland; ref no: 186002321) were used for the purification and concentration of HLA-I and HLA-II peptides. The C18 sorbents were conditioned, and the HLA complexes and bound peptides were directly eluted from the affinity plate with 1% trifluoroacetic acid (TFA; Sigma-Aldrich, Buchs, Switzerland). After washing the C18 sorbents with 0.1% TFA, HLA-I peptides were eluted with 28% acetonitrile (ACN; Sigma Aldrich, Buchs, Switzerland) in 0.1% TFA, and HLA-II peptides were eluted with 32% ACN in 0.1% TFA. Recovered HLA-I and -II peptides were dried using vacuum centrifugation (Concentrator plus, Eppendorf, Basel, Switzerland) and stored at –20 °C prior to MS analysis. Before injecting into the MS, the peptides were resuspended in 12 μL of 0.1% formic acid (FA) and 2% ACN, and 3 μL were injected per replicate.

### 2.6. LC-MS/MS Analyses

The LC-MS/MS system consisted of an Easy-nLC 1200 (Thermo Fisher Scientific, Waltham, MA, USA) coupled online to a Q Exactive HF-X mass spectrometer (Thermo Fisher Scientific, Waltham, MA, USA). Peptides (3 μg) were separated on a 450 mm analytical column (8 µm tip, 75 µm inner diameter, PicoTipTM Emitter, New Objective, Woburn, MA, USA) packed with ReproSil-Pur C18 (1.9 µm particles, 120 Å pore size, Dr. Maisch GmbH, Ammerbuch, Germany). The separation was performed at a flow rate of 250 nL/min using a gradient of 0.1% formic acid (FA) in 80% ACN and 0.1% FA in water. HLA-I peptides were separated for 120 min, HLA-II peptides for 90 min, and proteome digests were separated for 270 min.

The mass spectrometer operated in data-dependent acquisition (DDA) mode for both immunopeptidomics and proteomic samples measurement. For the measurement of HLA peptide samples, full MS spectra were acquired in the Orbitrap from m/z = 300–1650 with a resolution of 60,000 (m/z = 200) and an ion accumulation time of 80 ms. The auto gain control (AGC) was set to 3e6 ions. MS/MS spectra were acquired in a data-dependent manner on the 10 most abundant precursor ions (if present) with a resolution of 15,000 (m/z = 200), an ion accumulation time of 120 ms and an isolation window of 1.2 m/z. The AGC was set to 2e5 ions, the dynamic exclusion was set to 20 s, and a normalized collision energy (NCE) of 27 was used for fragmentation. No fragmentation was performed for HLA-I peptides with assigned precursor ion charge states of four and above, and for HLA-II peptides with an assigned precursor ion charge state of one, or six and above. The peptide match option was disabled.

For proteomic samples, full MS spectra were acquired in the Orbitrap from m/z = 300–1700 with a resolution 60,000 (m/z = 200) and an ion accumulation time of 80 ms. The AGC was set to 3e6 ions. MS/MS spectra were acquired on the 15 most abundant precursor ions (if present) with a resolution of 15,000 (m/z = 200), an ion accumulation time of 80 ms and an isolation window of 1.4 m/z. The AGC was set to 2e5 ions, the dynamic exclusion was set to 30 s and an NCE of 27 was used for fragmentation. No fragmentation was performed for an assigned precursor charge state of one. No MS internal standards were used. Treated samples were compared to control samples and a label free quantification (LFQ) algorithm implemented in Maxquant was used for comparative protein expression analyses [23].

### 2.7. Peptide and Protein Identification and Bioinformatic Analyses

We employed the MaxQuant computational proteomics platform version 1.5.5.1 [24] to search the MS peak lists against the UniProt databases (Human 42,148 entries, March 2017) and a file containing 247 frequently observed contaminants. All searches were performed with ‘match between runs’ option enabled and with the default settings unless explicitly specified. Processing of the output table was done with Perseus software [25]. ‘Dependent peptide’ search was performed on shotgun proteomics measurements done with quadruplicate A375 pellet, Ox-L and FT-L samples. PSMs with mass shift were retrieved from the ‘msmsScans.txt’ table.

Another MaxQuant analysis of shotgun proteomics was performed to assemble data of 3 independent biological experimental replicates of A375 pellet, Ox-L and FT samples (measured in triplicates or quadruplicates) together with two biological experimental replicates of immature and mature mo-DC loaded with A375 Ox-L, FT-L, or unloaded (measured in triplicates). Here, the label free quantification (LFQ) module was enabled and the following variable modifications were included: acetyl (protein N-term), carbamidomethyl on Cys residues, Trp oxidation to kynurenine, and oxidation on Cys, Lys, Met, Pro, Trp, and Tyr. The ‘modificationSpecificPeptides.txt’ output table was use to report PTM events, and the LFQ-protein intensities from the ‘ProteinGroups.txt’ output table were used for differential protein expression analyses. Entries matching to reverse and contaminants were filtered out. The values of peptide intensities or protein intensities were log2 transformed (Tables S2–S4) and then grouped together according to experimental replicates and conditions. Intensities were then filtered for at least 3 valid values in at least one condition. Missing intensity values were imputed by drawing random numbers from a Gaussian distribution with a standard deviation of 20% in comparison to the standard deviation of measured protein abundances. Volcano plots of changes in the relative LFQ intensities of identified proteins were created; significant events passed the selection criteria of *p* value < 0.05 and S0 = 1.

For immunopeptidomics analyses, protein FDR was set to 1 and the enzyme specificity was set as unspecific. First, HLA-I and HLA-II ligands purified from A375 pellet, FT-L and OX-L in triplicates and measured in technical duplicates were analyzed. The ‘peptide.txt’ output table was used. Entries matching to reverse and contaminants were filtered out. The values of peptide intensities were log2 transformed (Table S5) and then grouped together according to the conditions. Next, MS raw files of HLA-I and HLA-II ligands purified from immature mo-DCs, mature mo-DCs unloaded and mature mo-DCs loaded with either A375 Ox-L or A375 FT-L were analyzed in a separate MaxQuant analysis (Table S6). Here, the following modifications were included: oxidations on Lys, Met, Pro and Trp. In both immunopeptidomics datasets, peptide intensities were normalized using ‘width normalization’ option in Perseus, and missing intensity values were imputed by drawing random numbers from a Gaussian distribution with a standard deviation of 20% in comparison to the standard deviation of measured peptide abundances. Volcano plots of changes in the relative intensities of identified peptides were created; significant events were passed the selection criteria of *p* value < 0.05 and S0 = 1.

To estimate the solvent accessibility and surface exposure of peptides identified in the immunopeptidomic profiling, each peptide was first located in the 3D structure of its source protein retrieved from the Protein databank [26], as previously described [27]. Peptides identified included 9′668 and 10′431 peptides, respectively present in FT-L and Ox-L conditions in all the 3 biologic replicates. Among them, 4920 and 5365 peptides have structural representation in the source protein, for which we were then able to calculate solvent accessibility and surface exposure. For each of these peptides’ residues, residues solvent accessibility defined as the relative solvent excluded surface area (SESA) computed with the MSMS package, using the Chimera software [28] as described in [29], was then calculated. Finally, the relative SESA were calculated normalizing the surface area of the peptide of interest in its protein of origin by the surface area of the same isolated peptide in a reference state, as:

(1) $$\mathrm{SESA}=\frac{\sum \mathrm{Residue}.\mathrm{area}.\mathrm{SES}}{\sum \mathrm{Residue}.\mathrm{area}.\mathrm{SES}.\mathrm{gxg}}$$

where Residue.area.SES corresponds to the surface area of the individual residue in the protein of origin and Residue.area.SES.gxg corresponds to the surface area values per residue in a GLY-X-GLY tri-peptide, where X is the residue of interest, calculated as described previously in [30]. When a peptide could be found in several proteins, its SESA was calculated as the average of the solvent accessibility measured from all 3D structures of the putative proteins.

### 2.8. T Cell Phenotypic and Functional Analysis

Details for the clinical trial and vaccination schedule have been previously reported [16,17]. For the analyses here reported, cryopreserved peripheral blood mononuclear cells (PBMC) from pre- and post-vaccine (EOS) timepoints were thawed, rested overnight in RPMI with 10% FBS with Penicillin/Streptomycin at 37 °C and 5% CO_2_. The following day, moDCs loaded with HOCl-treated autologous tumor lysates were thawed and immediately added to autologous PBMCs at a ratio 1/10 in presence of 1 μg of brefeldin A (golgiplug, BD Biosciences, Allschwil, Switzerland; 555029) for an overnight incubation at 37 °C and 5% CO_2_. Cells were then washed and extra-cellularly stained with anti-CD3 (Biolegend, San Diego, CA, USA; 344840), anti-CD8 (Biolegend, San Diego, CA, USA; 301042), anti-CD4 (BD Biosciences, Allschwil, Switzerland; 558116), and a viability dye (Zombie UV, Biolegend 423107). After a permeabilization step, cells were intracellularly stained with anti-Granzyme B (Invitrogen GRB17), anti-Perforin (Biolegend, San Diego, CA, USA; 353310), anti-IL-2 (BD Biosciences, Allschwil, Switzerland; 559334, anti-TNF-α (BD Biosciences, Allschwil, Switzerland; 557647), anti-IFN-γ (BD Biosciences, Allschwil, Switzerland; 554702), anti-CD137 (4-1BB) (Biolegend, San Diego, CA, USA; 309808), and anti-CD154 (CD40L) (Biolegend, San Diego, CA, USA; 310826). The samples were acquired on a 5-laser BD Fortessa instrument equipped with the FACS Diva software (BD Biosciences, Allschwil, Switzerland). The analysis was performed with the FlowJo v10.5 ((BD Biosciences, Allschwil, Switzerland)) and SPICE 5.1 software (National Institutes of Health, Bethesda, MD, USA).

### 2.9. Statistics

Statistical analyses were performed with the Perseus computational platform version 1.5.5.3 (84) * *p* < 0.05; ** *p* < 0.01; *** *p* < 0.001; and **** *p* < 0.0001 were considered statistically significant.

### 2.10. Data Availability

MS RAW data, MaxQuant parameters, and the MaxQuant output tables used for analyses are available upon request to the corresponding authors.

## 3. Results

### 3.1. HOCl Incubation Induces Extensive Amino Acid Oxidation in Tumor Cells

**Figure 1:**
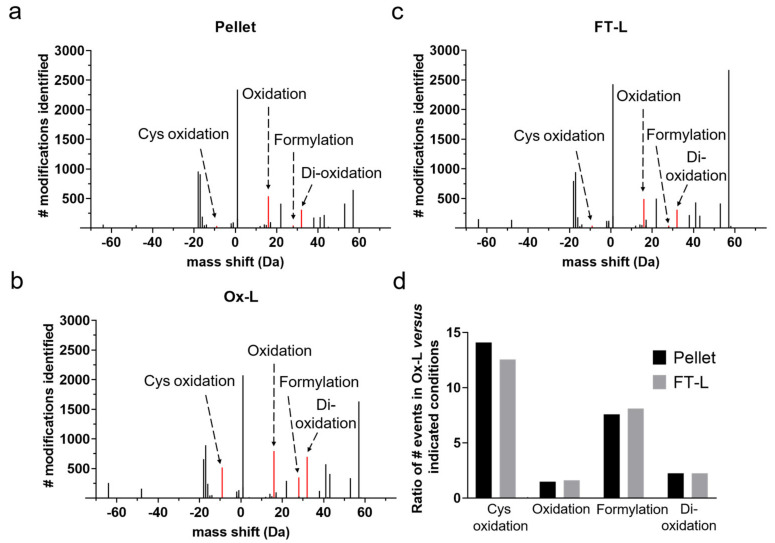
HOCl induces protein oxidation in cancer cells, in vitro. MaxQuant dependent peptide search results of proteomics analysis of A375 melanoma tumor cells that were incubated for 1 h in the presence (**a**) or absence (**b**) of 60 μM HOCl, washed and subjected to 6x freeze-thaw cycles, or left untouched after harvesting (**c**) The average number of mass shifts events compared to the unmodified peptides identified across technical replicates (*n* = 4) were reported; red lines represent the indicated specific modifications that increased significantly upon HOCl treatment. (**d**) Bar graph reporting the ratio of modifications identified in Ox-L over the indicated conditions.

In order to map modifications at the amino acid level induced by HOCl oxidation, we followed an optimized protocol previously used by our group in clinical vaccination studies, by incubating for one hour A375 melanoma tumor cells in the presence of HOCl, followed by six cycles of freeze/thaw to generate the WTL [16,31,32]. These studies showed, in fact, that using this WTL preparation as an antigen source efficiently increased tumor antigen uptake by mo-DCs and vaccine-induced tumor protective effects, compared to the not-oxidized control counterpart [16,31,32]. We then subjected this lysate (Ox-L) to shotgun proteomic analysis by mass spectrometry (MS). As controls, we analyzed in parallel, cells only subjected to freeze/thaw cycles (FT-L), or an untouched cell pellet (pellet). At first, in order to undertake a non-biased approach, we computationally analyzed the MS proteomics datasets with the ‘dependent peptide’ Andromeda search algorithm implemented in MaxQuant [33]. This search can identify peptides with amino acid containing post-translational modifications (PTMs), without specifying a priori mass shifts and amino acids involved. We observed obvious differences in PTMs between Ox-L and the control counterparts at the level of amino acid oxidation (Figure 1a–c). In particular, the number of peptide spectrum matches (PSMs) with single oxidation (mass shift: +15.99 Da) or double oxidization (mass shift: +31.99 Da) were significantly higher in Ox-L compared to the FT-L and pellet conditions, with increased ratios spanning from 1.5 to 2.2 (Figure 1d). Peptide amino acid formylation (mass shift: +27.99 Da) was also largely upregulated in Ox-L, compared to the pellet and the FT-L control counterparts with a ratio for the total number of formyl-containing peptides of 7.6 and 8, respectively (Figure 1d). In addition to this, Cys oxidation was significantly more detected in Ox-L too (Figure 1d). In particular, this modification was characterized by a specific mass shift of –9.03 Da due to the expected presence of a carbamidomethyl moiety on free Cys, as a consequence of Iodoacetamide treatment during sample preparation (Figure 1a–c). Regarding the specific oxidation of tryptophan to kynurenine, we observed an increase in Ox-L compared to both FT-L and pellet conditions, however the number of identified PSM events in these settings was rather low (<20 events).

**Figure 2:**
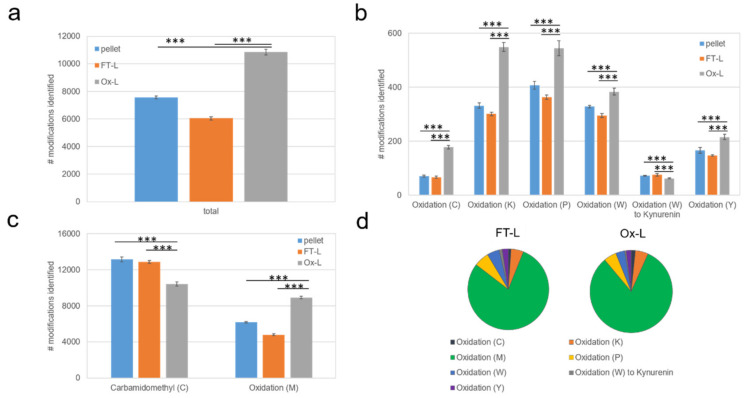
HOCl increases post-translational modifications on Met, Lys, Pro, Cys Trp and Tyr in cancer cells, in vitro. Proteomics results including the oxidations as variable modifications. (**a**) Bar plot reporting the average number of oxidized Met, Lys, Pro, Cys, Trp and Tyr residues ± standard deviation identified in the indicated conditions (*n* = 4 technical replicates). (**b**) Same as (a) with each oxidized modification reported separately. (**c**) Bar plot reporting the average number of Carbamidomethyl modifications on Cys residues and of oxidized Met residues ± standard deviation identified in the indicated conditions (*n* = 4 technical replicates). (**d**) Distribution of total number of oxidative modifications identified on average in indicated samples. Data were analyzed with unpaired student t-test: *** p < 0.001.

Based on these results, we decided to include Cys-, Lys-, Met-, Pro-, Trp-, Tyr-oxidation, and kynurenine (Kyn) formation as potential variable modifications for a subsequent MaxQuant peptide search analysis. In addition to this, due to the observation of extensive Cys oxidation (Figure 1d) we also considered the presence of a carbamidomethyl moiety on free Cys residues as a variable modification to increase the sensitivity of the peptide search for Cys oxidation. With these settings, we performed a second proteomic analysis of the A375 melanoma lysate treated with HOCl (Ox-L), untreated (FT-L) or untouched cells (pellet) over three biological (i.e., experimental) replicates and for subsequent analyses we report the more consistent experimental replicate. Analysis of the A375 proteome dataset (Figure S1) identified a total of 4920 proteins of which 4610 were present in all three groups of samples. We observed extensive peptide oxidation upon HOCl treatment, with an average of 10,850 oxidized events in Ox-L, compared to 6045 and 7563 in the FT-L and pellet controls, respectively (Figure 2a). In particular, the mostly occurring oxidized amino acids in the Ox-L condition were in the order: Met≫Lys>Pro>Trp>Tyr>Cys>Kyn (Figure 2b,c). Interestingly, this observation is consistent with previous in vitro studies showing that Met is the amino acid most prone to HOCl oxidation in vitro, in physiological conditions [34,35]. Importantly, all oxidized amino acid residues included in the search occurred significantly more in the HOCl-oxidized lysate, except for Kyn, a byproduct of Trp oxidation [36], while the average ratio on the total number of events for each modification spanned between 1.2 and 2.7, when comparing Ox-L to controls (Figure 2b,c). As expected, concomitantly with an increase on Cys oxidation, we also observed a corresponding distinct decrease in carbamidomethyl occurrence at Cys residues in Ox-L, compared to both controls (Figure 2b,c). On the other hand, the global distribution of oxidative modifications across the different amino acids taken into consideration did not substantially change upon HOCl incubation (Figure 2d).

In conclusion, our results demonstrated that the major effects of HOCl in tumor cells, at the level of PTMs and protein composition, consists in amino acid oxidation, with Met being by far the mostly affected residue. This in line with previous evidence suggesting that Met is the most readily oxidized residue in proteins [34,37]. Thus, HOCl treatment induces extensive protein oxidation, whilst maintaining an unvaried global distribution among the different amino acids amenable of oxidation.

### 3.2. HOCl Induces Specific Patterns of Protein Expression and Oxidation in Tumor Cells

**Figure 3:**
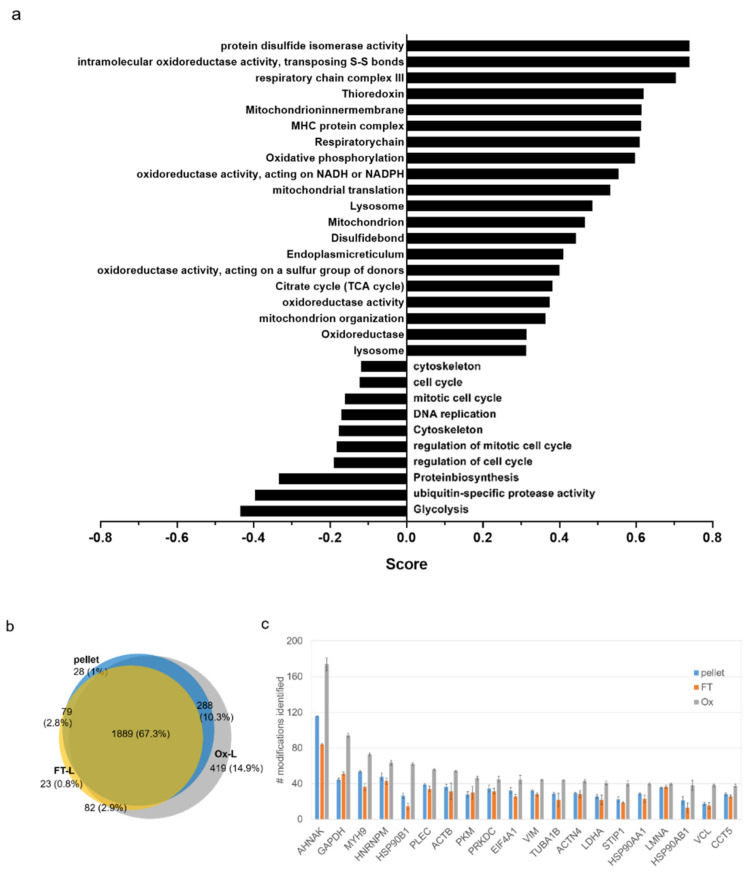
HOCl induces specific patterns of protein oxidation and expression in melanoma cancer cells. (**a**) Significant fold change trends in Ox-L compared to FT-L, obtained from gene ontology enrichment analysis (*p* < 0.05). (**b**) Venn diagram reporting the number of oxidized proteins that overlap between pellet (blue circle), Ox-L (grey circle) and FT-L (orange circle) conditions. (**c**) Average number of oxidized residues in the indicated proteins and conditions ± standard deviation (*n* = 4 technical replicates).

We further explored whether HOCl induces significant quantitative changes at the proteome level in tumor cells. In order to specifically single out the effect of HOCl treatment, we focused on the comparison between the Ox-L and the FT-L group samples. We identified 926 proteins significantly more abundant and 946 less abundant in the Ox-L condition, compared to FT-L (test FDR = 0.05 and S0 = 1, Figure S1c). Interestingly, a gene ontology analysis revealed an upregulation on protein folding, and mitochondrial components, a downregulation of proteins involved in cell cycle, as well as a concomitant metabolic shift from glycolysis to citrate cycle and oxidative phosphorylation (OXPHOS) (Figure 3a). As expected, these changes point out that cell incubation with HOCl induces extensive oxidative stress leading to mitochondrial damage [38] and dysfunction [39], cell cycle arrest (probably due to DNA damage), protein oxidation, and unfolding. As a consequence of this, tumor cells responded by activating the unfolded protein response machinery (e.g., increased expression of heat shock proteins (HSPs) [40]), inducing mitochondrial biogenesis [41] and upregulating anti-oxidant proteins (e.g., thioredoxins [42], disulfide isomerases [43]) in an attempt to restore redox balance and homeostasis.

Interestingly, a protein-centric analysis showed that more than 400 proteins (14.9% of the totally identified proteome) were uniquely oxidized (in at least one amino acid residue) in A375 cells upon HOCl incubation (Figure 3b). Furthermore, among the other commonly oxidized proteins present in all three samples Ox-L, FT-L, and pellet, the total number of oxidation sites/protein varied largely according to the condition tested. An analysis of the 20 top proteins containing the highest number of oxidized residues revealed that these were all significantly more oxidized upon HOCl treatment (in terms of total number of oxidized residues), compared to the FT-L and pellet counterparts (Figure 3c). In particular, when we considered proteins with at least five oxidized residues, we identified 477 proteins containing significantly more oxidized residues in the Ox-L condition, compared to the two controls. Instead, no protein in the whole data set was significantly more oxidized in the FT-L or pellet, compared to Ox-L. Interestingly, a large proportion of heavily oxidized proteins in the Ox-L condition corresponded to highly abundant proteins, previously shown to be prone to oxidation such as cytoskeletal proteins (vimentin [44], tubulin [45]) or proteins upregulated in response to oxidative stress (e.g., HSPs) [40], calreticulin [46]). In addition to this, a subset of heavily oxidized proteins in Ox-L were also previously reported to be involved in their oxidized form in common oxidative stress response pathways such as nuclear lamins [47], glyceraldehyde 3-phosphate dehydrogenase (GAPDH) [48], and actin [49].

### 3.3. HOCl Antigen Treatment Does not Significantly Affect DC Phenotype

**Figure 4:**
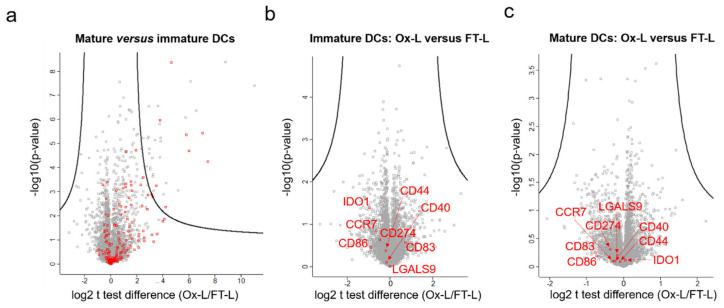
HOCl antigen treatment does not influence DC phenotype and maturation status. Mo-DCs were loaded with Ox-L, FT-L or left unloaded (antigen negative control) for 24 h, then incubated either with LPS and IFN- (mature DCs) or with no supplements (immature DCs). Cells were then extensively washed, trypsinized and analyzed through LC-MS/MS. (**a**) Volcano plot representation of t test analysis comparing protein intensities in mature versus immature mo-DCs; each point represent one protein plotted by log2 fold change versus minus logarithm of the *q*-value (Benjamini-Hochberg corrected *p*-value), with a cutoff value of 0.05; in red genes belonging to the immunity class, expected to be upregulated upon DC maturation (keywords classification). (**b**) Same as (a) comparing immature DCs loaded with Ox-L versus FT-L, in red genes encoding for main surface proteins with stimulatory or inhibitory effects on T cell activation. (**c**) Same as (**a**) comparing mature DCs loaded with Ox-L versus FT-L, in red genes encoding for main surface proteins with stimulatory or inihibitory effects on T cell activation.

Whether HOCl oxidation of antigen(s) improves mo-DC maturation and ligand surface exposure still remains a controversial aspect in the field with so far contradictory reports [10,12,13]. Thus, to further address this point, we first generated immature mo-DCs from two distinct healthy donors, loaded them with Ox-L, FT-L, or kept them unloaded (as an antigen negative control) and subsequently matured them in the presence of IFN- and liposaccharide (LPS), as previously described [16]. We then performed a proteomic analysis of these samples to potentially identify global protein expression changes. As expected, a significant upregulation of proteins belonging to the immunity class (according to keywords classification, *p* < 0.001) was observed upon mo-DC maturation (Figure 4a). However, no significant changes in protein expression levels were identified when comparing mo-DCs loaded with Ox-L or FT-L, both at the immature state (Figure 4b) or after DC maturation (Figure 4c). Specifically, no significant difference was observed for co-stimulatory molecules such as CD40, CD80, CD86, and OX40-Ligand essential for T cell stimulation, or proteins with T cell inhibitory activity such as IDO, Galectin-9, or CD274. Interestingly, despite the increased presence of oxidized residues in the HOCl-treated cell lysate, compared to the untreated one (Figure 2), no specific enrichment for oxidized peptides in the loaded immature (Figure S2a) or mature (Figure S2b) DC proteome was observed when comparing DCs loaded with Ox-L or with FT-L, suggesting that the contribution of lysate-derived proteins at the DC proteome level is negligible, at least in this respect (*p* > 0.05 in both cases). In addition to this, to evaluate and compare the antigen processing machinery in mo-DCs loaded with Ox-L or FT-L, we specifically analyzed in the same data set the levels of proteins known to be involved in antigen processing and presentation [50,51] and generated a heat map. As expected, the obtained sample clustering clearly showed that the different tested conditions clustered based on their maturation state (matured versus immature clusters) or based on the belonging donor (donor 1 versus donor 2) (Figure S3). However, no significant clustering or differentiation was highlighted based on the antigen source (FT versus Ox-L), confirming that reported above at the whole proteome level (Figure 4b,c).

Overall, these results suggest that the HOCl-induced improved immunogenicity is not related to a change in the DC phenotype, to enhanced expression of co-stimulatory molecules (e.g., CD86, CD83), or to differential antigen processing and presentation capabilities, at least in our experimental conditions.

### 3.4. HOCl Antigen Treatment Significantly Improves MHC-II Antigen Presentation in Mo-DCs

Next, in order to assess the influence of HOCl treatment on the HLA presentation and the antigenic repertoire of loaded mo-DCs, we performed an immunopeptidomic analysis on mo-DCs loaded with A375-derived Ox-L or FT-L, as antigen source or left unloaded as an antigen negative control. In this analysis, we sequentially immunoaffinity-purified HLA-I and HLA-II complexes from mo-DCs, extracted the HLA binding peptides and measured the peptides by mass spectrometry (see Methods section) [52]. In order to rule out any significant direct contribution of residual HLA-peptide complexes originating from A375, we first performed an immunopeptidome analysis of the A375 tumor cell lysate, either previously treated with HOCl (Ox-L) or left untreated (FT-L), to assess the stability of the HLA complexes upon treatment. Contrary to expectations, we detected stable complexes in the tumor cell lysate, after HOCl incubation and lysate preparation. In particular, from HLA-I complexes we identified 8618 unique peptides in total, with 67.7% of them in common among the three tested conditions (Figure S4a), and an overall comparable number of peptides isolated from FT-L and Ox-L conditions (Figure S4b). For HLA-II complexes, we identified 5433 unique peptides in total with only 21.1% overlap among the three analyzed conditions (Figure S4c). In this case, the number of peptides isolated from Ox-L were significantly lower compared to the other two control counterparts, even though still considerably present (Figure S4d).

Finally, as a further validation, we also applied the previously developed Gibbs algorithm [53] to our peptide data set, in order to identify recurring binding motifs and ultimately compare them with the ones corresponding to the known cell line haplotype (Table S1). Our results unambiguously showed that the recurrent HLA binding motifs from our peptide dataset (Figure S4e) largely aligned with the expected motifs of the cell line haplotype [54]. This include for example the presence of the aromatic residues Trp and Phe in position 9 corresponding to HLA-B*57:01, HLA-C*06:02, and HLA-C*16:02 common binding motifs, as well as Asp in position 2 and Tyr in position 9, corresponding respectively to the HLA-A*01:01 and HLA-B*44:03 binding motifs [54]. These observations further confirmed the experimentally identified peptides as bona fide ligands for the corresponding cell line HLA alleles.

Taken altogether, these results demonstrated that the HOCl incubation and lysate preparation did not completely disrupt HLA-peptide complexes, at least in the A375 melanoma cell line here analyzed. Therefore, in order to minimize potential contaminating HLA complexes and to allow a direct assessment of HLA presentation taking place endogenously at the DCs level by the DC antigen processing machinery, we specifically selected a healthy donor with a mismatched HLA haplotype compared to the A375 cell line for mo-DCs preparation (Figure S4 and Table S1). Application of the Gibbs algorithm [53] to the resulting mo-DCs HLA-I peptide data set confirmed the correct clustering of detected peptides only with the healthy donor HLA haplotype and not with the cell line haplotype, confirming the overall exclusion of peptides derived from residual tumor cell HLA-peptide complexes or from peptide exchange (data not shown).

**Figure 5:**
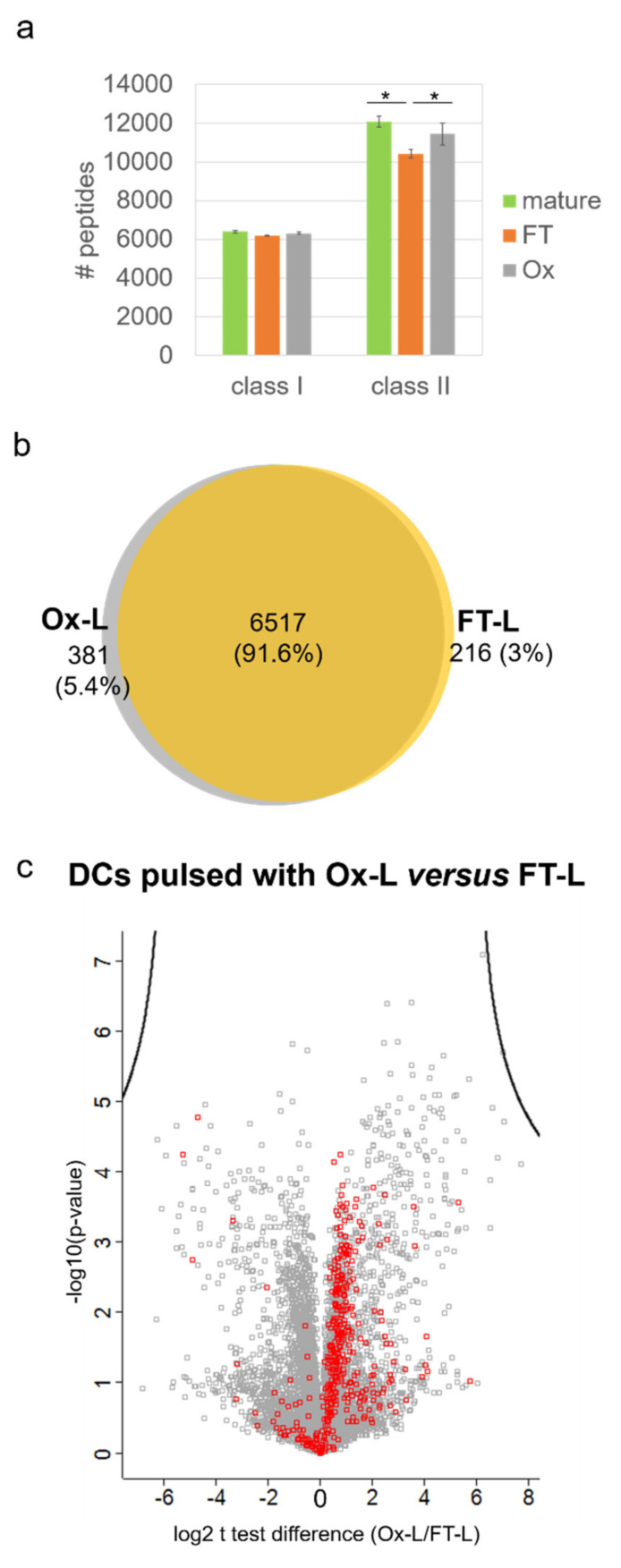
HOCl antigen treatment does not significantly impact on HLA class-I antigen presentation in mo-DCs. Mo-DCs from a healthy donor were loaded with Ox-L or FT-L, or left unloaded (antigen negative control) and subsequently matured in the presence of IFN-γ and LPS. HLA-I and –II peptide were extracted and analyzed by LC-MS/MS. (**a**) Average number of unique peptides isolated from HLA-I and –II complexes from mo-DCs loaded with the indicated antigens (*n* = 3 technical replicates). (**b**) Venn diagram reporting the overlap between the number of unique peptides isolated from HLA-I complexes from mo-DCs loaded with the indicated antigen source. (**c**) Volcano plot representation of t test analysis comparing HLA-I peptide intensities in mo-DCs loaded with Ox-L versus FT-L; each point represent one peptide plotted by log2 fold change versus minus logarithm of the *q*-value (Benjamini-Hochberg corrected *p*-value), with a cutoff value of 0.05; red squares indicate peptide containing ox-Met residues. * *p* < 0.05.

Concerning the HLA class I peptide repertoire of mo-DCs loaded with A375-derived Ox-L or FT-L, or left unloaded, results showed that the average number of peptides isolated from the three tested conditions were essentially comparable (Figure 5a), and their identity was overall conserved with a ~92% overlap among unique peptides identified in the FT-L and Ox-L conditions (Figure 5b). Interestingly, when analyzing peptide intensities in terms of fold change, we were not able to identify any peptides preferentially presented by mature mo-DCs loaded with Ox-L, compared to FT-L (Figure 5c). More specifically we found no significant changes in peptides belonging to known validated cancer antigens such as survivin [55], NY-ESO [56] or melanoma-associated antigen protein family genes (MAGEs) [57] that could have partially explained the increased immunogenicity, previously observed upon HOCl antigen treatment [16]. On the other hand, we clearly observed a trend of increased presentation of peptides containing oxidized Met (ox-Met) in Ox-L compared to the FT-L condition (*p* < 0.001) (Figure 5c). This may reflect the higher abundancy of ox-Met-containing proteins in the antigen source following HOCl treatment, as reported above (Figure 2c).

**Figure 6:**
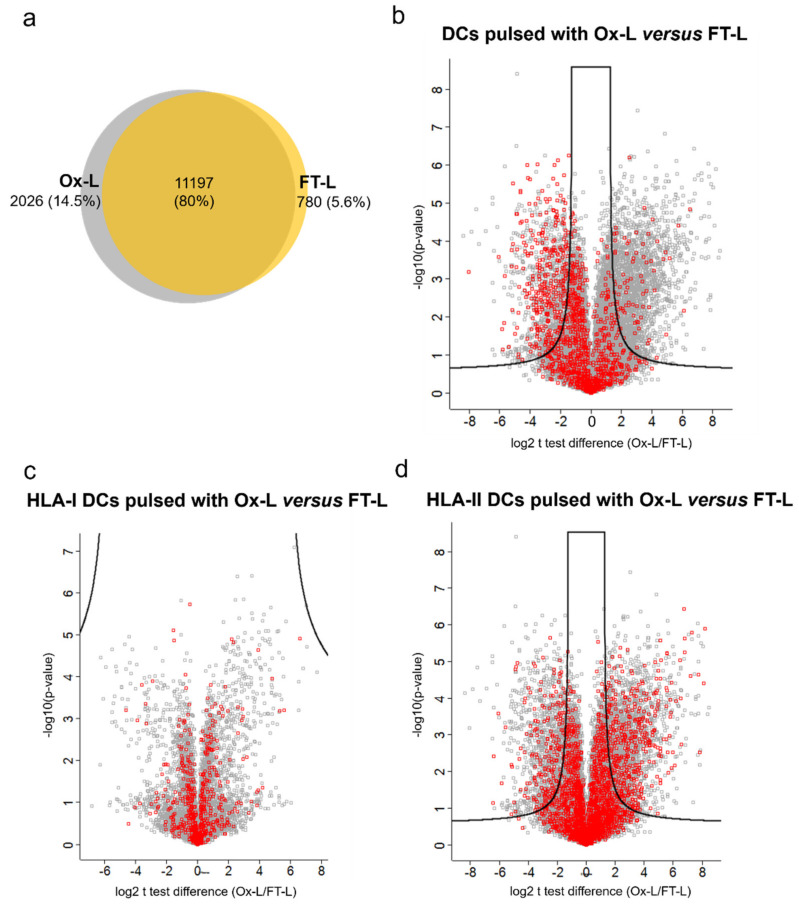
HOCl antigen treatment significantly increases HLA class-II antigen presentation in mo-DCs. Samples were prepared and analyzed as reported in Figure 5. (**a**) Venn diagram reporting the overlap between the number of unique peptides isolated from HLA-II complexes from mo-DCs loaded with the indicated antigen source. (**b**) Volcano plot representation of t test analysis comparing HLA-II peptide intensities in mo-DCs loaded with Ox-L versus FT-L; each point represent one peptide plotted by log2 fold change versus minus logarithm of the *q*-value (Benjamini-Hochberg corrected *p*-value), with a cutoff value of 0.05; red squares indicate peptide containing ox-Met residues. (**c**,**d**) Volcano plot representation of t test analysis comparing HLA-I (**c**) and HLA-II (**d**) peptide intensities in mo-DCs loaded with Ox-L versus FT-L; each point represent one peptide plotted by log2 fold change versus minus logarithm of the *q*-value (Benjamini-Hochberg corrected *p*-value), with a cutoff value of 0.05; red squares indicate peptides from putative proteins significantly upregulated in Ox-L compared to FT-L (from Figure S1).

Interestingly, more distinct patterns emerged concerning the HLA-II peptide repertoire. First of all, 2026 peptides were uniquely presented in mo-DCs loaded with Ox-L, compared to FT-L, while only 780 were uniquely identified in the FT-L condition (Figure 6a). Furthermore, when comparing the intensities of peptides isolated from mo-DCs loaded with Ox-L or FT-L, we noticed a clear shift towards higher peptide abundance in the former case (Figure 6b), which was confirmed by a significant increase in the average peptide intensity (*p* < 0.001). Surprisingly, when analyzing the trends of oxidized peptides, we observed a significant decrease for ox-Met-containing peptides in the Ox-L sample compared to FT-L (Figure 6b).

Next, to further dissect the immunogenic role of HOCl, we analyzed the levels of presentation of peptides from the 926 proteins that were found to be significantly upregulated in the proteomics analysis upon HOCl antigen treatment in Ox-L, compared to FT-L. Among these, we identified peptides from 343 of them in the mo-DC HLA-I ligandome. However, these peptides were overall equally presented when comparing mo-DCs loaded with Ox-L or FT-L (Figure 6c). On the other hand, in the case of HLA-II-derived peptides, we identified peptides from 268 upregulated proteins with a significant trend towards higher abundance in mo-DCs loaded with Ox-L compared to FT-L (*p* < 0.05) (Figure 6d).

### 3.5. Solvent Exposed Regions in Source Proteins upon HOCl Treatment Contribute to the Mo-DCs HLA-II Ligandome

**Figure 7:**
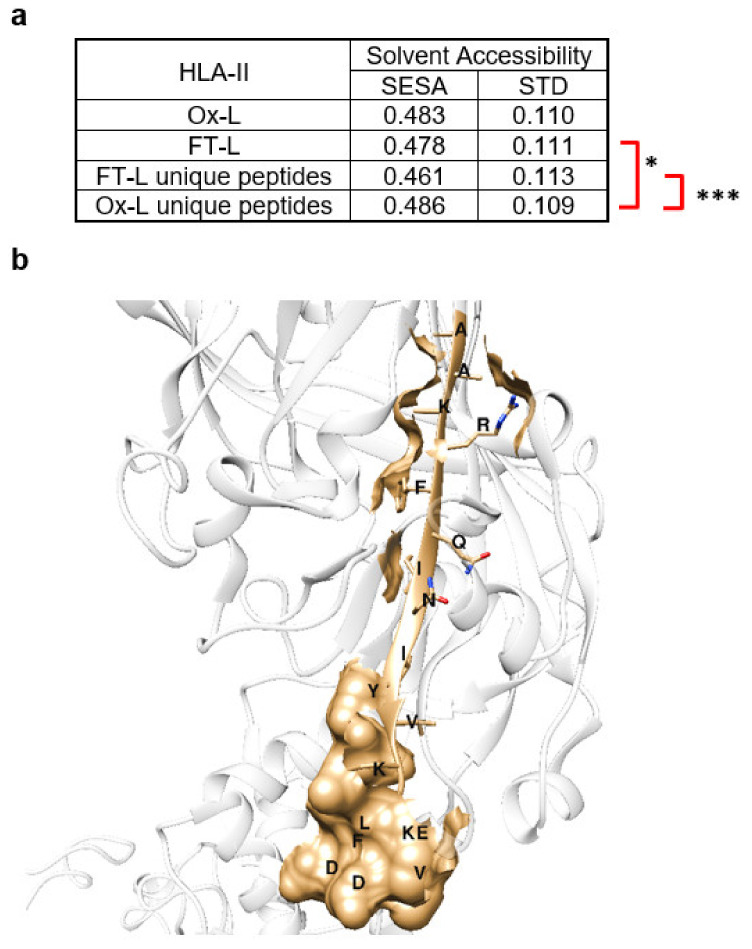
Analysis of solvent exposure for HLA-II ligand peptides indentified in this study in their source proteins. (**a**) Average solvent accessibility (SESA) for HLA-II ligand peptides isolated from mo-DCs loaded with the indicated conditions; *unique peptides* indicates peptides only identified in the indicated condition. SESA values were estimated by mapping each peptide on the 3D structure of the source protein and then calculating its surface exposure; statistical analysis: * *p* < 0.05; *** p < 0.001 (**b**) Surface exposure representation of the peptide AAKRFQINIYVKKLDDFVE, belonging to lysosome membrane protein 2 (residues 379.A–397.A) with a calculated SESA value of 0.430; protein structure retrieved from the Protein Data Bank: 4f7b, as an example.

It has been previously speculated, that unique epitopes presented by DCs upon HOCl antigen treatment result from a higher protein susceptibility to proteolysis due to oxidation and subsequent protein unfolding, leading to the increased exposure of otherwise less accessible sites [9,13]. To verify this hypothesis within our data, we analyzed and compared the solvent exposure of the identified HLA-II peptides from both the Ox-L and FT-L conditions. To this aim, we mapped each peptide in the 3D structure of their respective putative source protein and estimated its solvent exposure by calculating the relative solvent excluded surface area (SESA), as previously reported [27,29]. Of note, despite the fact that SESA calculations were not possible for all the identified peptides due to the lack of a reported 3D structure, analyzed peptides were a representative subset of the MHC-II ligands with equivalent peptide length distribution and amino acid frequencies (Figures S5 and S6). Obtained SESA values, which span from 0 for totally buried peptides to 1 for fully solvent exposed peptides, were then averaged for each peptide group (Figure 7a). A peptide with an average calculated SESA of 0.43 is reported in Figure 7b, as a representative example. Interestingly, a direct comparison between the average SESA of the HLA-II ligandome for the Ox-L condition and for the FT-L condition clearly showed that HOCl treatment had overall no significant impact (0.483 and 0.478, respectively). In addition to this and contrary to our expectation, when we compared the SESA average value for the FT-L condition with the one calculated only for peptides uniquely presented in the Ox-L condition, we actually observed a significant increase in the latter, from 0.478 to 0.486 (*p* = 0.0125). Hence, this evidence suggests that peptides uniquely presented upon HOCl treatment were on average more solvent exposed, contradicting the previously proposed hypothesis that unique epitopes arose from an HOCl-induced protein unfolding and an increase in the proteolytic activity of more deeply buried epitopes [9,13].

### 3.6. Vaccination with Mo-DCs Antigen-Stimulated with Autologous Ox-L Activates CD4+ T Cell Responses in Ovarian Cancer Patients, in Adjuvant Settings

**Figure 8:**
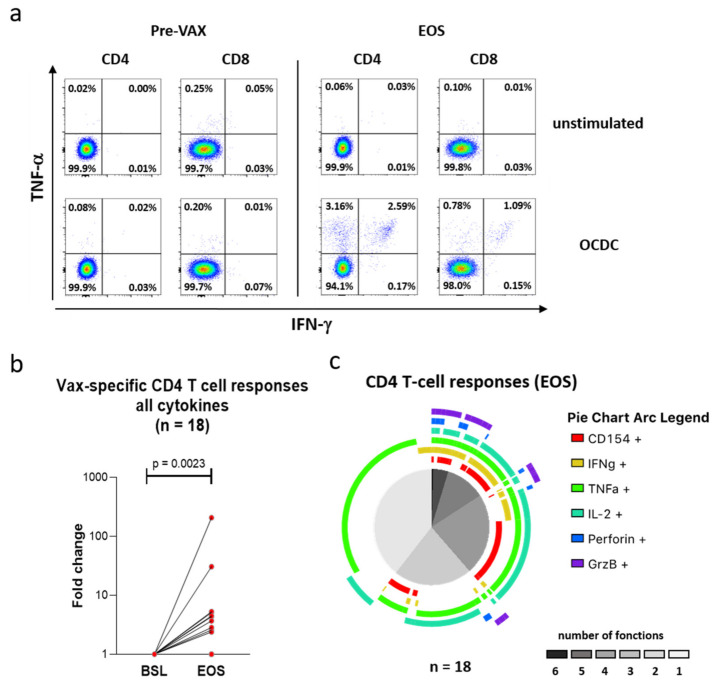
Patient vaccination with mo-DCs loaded with autologous Ox-L efficiently induces polyfunctional (i.e., the production of at least two cytokines) tumor- and neoantigen-specific CD4^+^ T cell responses. (**a–c**) Peripheral blood mononuclear cells (PBMC) were isolated from ovarian cancer patients vaccinated with mo-DCs loaded with autologous Ox-L (OCDC) either prior (Pre VAX) or after vaccination (EOS) and incubated in vitro with OCDC. T cell phenotype and cytokine production were assessed by intracellular cytokine staining followed by flow cytometry analysis (ICS). For the details of the vaccination schedule we refer to our previous publication [16,17].

As previously reported, our group recently conducted a Phase I clinical study in ovarian cancer patients that were vaccinated with moDCs loaded with HOCl-treated autologous tumor lysates (OCDC), demonstrating a significantly prolonged survival and the induction of *de novo* anti-tumor T cell responses [16,17]. In light of the observations reported above for an increased HLA-II presentation in mo-DCs loaded with HOCl-treated tumor lysates, we decided to specifically investigate the CD4^+^ T cell responses elicited by the OCDC vaccine in another subset of 18 patients treated under the same protocol. Interestingly, these analyses showed that the treatment was able to induce vaccine-specific CD4^+^ T cell responses, as demonstrated by the detection of cytokine-producing CD4^+^ T cells upon in vitro re-challenging with OCDC, from cells isolated after vaccination (compared to pre-vaccination levels) (example shown in Figure 8a). This amplification was in particular more pronounced in the CD4^+^ T cell compartment, compared to the CD8^+^ one (Figure 8a). Importantly, when patients’ CD4^+^ T cells were in vitro re-challenged with OCDC, the fold increase of cytokine-producing CD4^+^ T cells was significantly more pronounced after vaccination, compared to pre-vaccination baseline levels (Figure 8b). Finally, we comprehensively profiled vaccine-specific CD4^+^ T cells isolated after vaccination and observed significant polyfunctionality (i.e., the production of at least two cytokines) in more than 60% of the functional CD4^+^ T cells (Figure 8c). Taken altogether, these results further suggest that the CD4^+^ T cell activation was indeed vaccine-dependent and that using HOCl-treated tumor lysates as antigen source for therapeutic vaccination purposes leads to a stimulation of the CD4^+^ T cell compartment, at least in adjuvant settings in ovarian cancer patients.

## 4. Discussion

DCs are the most potent APCs in the human body and regulate both innate and adaptive immune responses against pathogens [1]. For these reasons, they have been long pursued in the context of cancer immunotherapy to mount a cancer-specific immune response [58]. However, despite their immunological crucial role, clinical therapeutic outcomes are still limited and considered largely improvable [6,7].

Previous work demonstrated that treating tumor cells with HOCl (a potent oxidant that is also present in physiological conditions at the early steps of an infection response [8]) induces cell necrosis [12]. In addition to this, treating the antigen source with HOCl increases crucial steps such as proteolysis [9,13], DC antigen uptake [12,13], and ultimately downstream immune responses [9,10,11,12,14,15,16], with even some distinct clinical benefits reported so far in ovarian cancer patients [16,17]. Cellular proteins are considered the major target of HOCl reactivity [35,59]; however, the mechanisms behind this HOCl-induced improved immunogenicity still remain elusive. To address this issue, we conducted a proteomic analysis of melanoma tumor cells treated with HOCl followed by lysate preparation following the same protocol preparation as in the clinical study [16,17], and compared it to an untreated lysate control. Further to this, we also investigated the effects of this lysate used as an antigen source to load mo-DCs from healthy donors, at both the DC phenotypic (proteomics) and the antigen presentation (immunopeptidomics) levels. As expected, results from tumor cell proteomics showed extensive protein oxidation, especially on Met residues, upon HOCl incubation. This is in line with previous studies reporting that Met is the most readily oxidized residue in proteins, which in physiological conditions is quickly attacked by oxidative agents (e.g., reactive oxygen species (ROS)) [34,60]. To overcome this and maintain redox balance and homeostasis, cells are nearly universally equipped with an enzymatic cascade that is able to restore Met in its reduced form, at the final expenses of NADPH [61,62]. In this context, it has been also recently proposed that Met residues present at the protein surface act as ROS scavengers and as a first line of antioxidant defense, ultimately protecting critical residues present in the enzymatic active site against detrimental oxidative modifications [37]. In light of this, it is not surprising, therefore, that Met is by far the most oxidized residue by HOCl, in our experimental system. Notably, among the list of heavily oxidized proteins following HOCl treatment we did not only find normally abundant proteins (e.g., cytoskeletal proteins such as vimentin and tubulin) previously shown to be susceptible of oxidation in the presence of ROS [44,45], but also proteins (such as nuclear lamins, GAPDH, and actin) that, once oxidized, play important protective roles against oxidative stress by activating counteracting mechanisms [47,48,49].

In addition to this, when looking at protein expression levels, we identified nearly 2000 proteins whose expression was significantly altered upon HOCl treatment in the A375 melanoma cell line. Importantly, when we examined this list through gene ontology enrichment analysis, we observed a clear upregulation in both the unfolded protein response and mitochondrial classes, together with a downregulation of proteins involved in cell cycle regulation and a metabolic reprogramming skewed towards OXPHOS. Such changes were likely involved in the tumor cell response against the extensive oxidative damage induced by HOCl, in an attempt to restore cellular homeostasis. In particular, among the list of significantly upregulated proteins, we observed several chaperones and HSPs with interesting immunogenic roles such as calreticulin, endoplasmin, Hsp10 and Hsp60, among others. These proteins are in fact not only generally classified as damage-associated molecular patterns (DAMPs) and important drivers of DC maturation [63,64,65,66,67], but few of them are also able to carry tumor-associated peptides and proteins, efficiently transfer them to DCs through cell surface HSP-specific receptors and ultimately promote their presentation [68]. Thanks to these adjuvant properties, this approach has been exploited by several groups in the design of Hsp-peptide-based vaccines and immunotherapies [69,70]. In light of this, the increased presence of HSPs upon HOCl incubation constitutes therefore an interesting feature that may well importantly contribute to the final increased immunogenicity experimentally observed.

Whether HOCl antigen treatment ultimately improves DC maturation and surface molecule expression has been long debated in the field, with so far few contradictory reports. Chiang et al. observed an increase in CD86 and CD40 surface expression in mo-DCs upon stimulation with HOCl-treated SKOV-3, however these levels were significantly lower than in DCs treated with LPS [12]. On the contrary, no increase in CD86 and MHC-II surface levels were recorded in two other independent studies, using model proteins [10,13]. However, it should be noted that such studies used different antigen sources and experimental conditions, which may partially explain the observed discrepancies. In our vaccine formulation, we extensively wash tumor cells after the incubation with HOCl to remove any residual HOCl that may subsequently affect DC viability, hence any effect on the DC phenotype is not directly due to the direct exposure to HOCl but rather to the intrinsic differences between Ox-L and FT-L described above. Nonetheless, in our experimental system we observed no significant effects that can be attributed to HOCl antigen treatment at the level of DC maturation or phenotypic protein expression, including in protein levels of antigen processing and presentation components. This evidence, together with the fact that we used mo-DCs from the same batch to load with either Ox-L or FT-L throughout the study, collectively suggest that the observed increased immunogenicity is not linked to a difference in the antigen processing and presentation machinery in mo-DCs loaded with Ox-L versus FT-L. However, future work should further investigate this aspect.

Finally, when analyzing both the HLA-I and HLA-II ligandomes of mo-DCs loaded with Ox-L or FT-L, a rather interesting scenario emerged. In particular, we observed that all significant trends and differences observed between the Ox-L and FT-L conditions concerned the HLA-II ligandome, while no significant changes were recorded when comparing the two HLA-I ligandomes. These included a significant increase in the total number of HLA-II ligands, their average intensities, and the number of unique peptides present in the Ox-L condition, compared to FT-L. This demonstrates that HOCl treatment increases MHC-II antigen presentation both from a qualitative point of view (higher number of unique ligands presented) and quantitative point of view (higher levels of ligands presentation). These results are in accordance with a recent report showing that HOCl treatment induces higher antigen-specific antibody production in a mouse model, indicative of a stronger MHC-II signaling [18].

Interestingly, when focusing on the solvent exposure levels of HLA-II epitopes uniquely presented in mo-DCs loaded with Ox-L we observed that these peptides were not on average more deeply buried in the protein core as previously speculated [9,13], but were actually more solvent exposed. This evidence suggests that the mechanism of action for HOCl-induced increased immunogenicity does not rely on the antigen processing and presentation of normally inaccessible core proteins epitopes that could become exposed in this case upon HOCl-induced protein unfolding. Importantly, changes in the tumor cell proteome induced by HOCl were well reflected also at level of the HLA-II presentation in which case peptides belonging to upregulated proteins in Ox-L were also overall significantly more abundant in the HLA-II ligandome of mo-DCs loaded with Ox-L compared to FT-L. Interestingly, we notice a trend of higher presentation of oxidized HLA-I peptides in mo-DCs loaded with Ox-L compared to FT-L, suggesting that the loaded proteins were degraded by two distinct cellular machineries for loading on either HLA-I or HLA-II. However, the exact mechanism needs to be further explored.

Our analysis demonstrated a CD4^+^ T cell activation induced in ovarian cancer patients vaccinated with autologous DCs loaded with HOCl-treated autologous tumor lysates in a phase I clinical study. Importantly, this CD4^+^ T cell activation was vaccine-specific and polyfunctional. This result complements previously published observations from our group on the polyfunctional and vaccine-specific CD8^+^ T cell activation induced by OCDC in the same set of patients [17]. Several studies previously showed the crucial importance of activating the CD4^+^ T cell compartment, in addition to cytotoxic CD8+ T cells, a requirement for the induction of spontaneous and immunotherapy-induced anti-tumour responses [71,72,73]. The evidence here presented suggest therefore that the success of using HOCl-treated tumor lysates may partially lie in the induction and activation of a polyfunctional CD4^+^ T cell response.

## 5. Conclusions

The evidence collected herein suggests that the increased immunogenicity experimentally observed when using cancer cells treated with HOCl as antigen source depends, at least partially, on extensive oxidative stress induced by HOCl, leading to subsequent changes in the cell proteome and the upregulation of key proteins (e.g., HSPs, mitochondrial components etc.) involved in the restoration of cell homeostasis. These changes are then reflected in the enhanced HLA-II antigen processing and presentation by loaded mo-DCs, while HLA-I presentation essentially remained unvaried, thus potentially leading to an increased HLA-II dependent stimulation of the CD4^+^ T cell compartment. Given the complex nature of the adaptive immune system, it is however clear that these mechanisms may well only significantly contribute, but not be solely responsible for, the experimentally observed increased immunogenicity linked to HOCl. Future work should further investigate the clear downstream effects of HOCl adjuvant properties at the levels of CD4^+^ T cells and B cells activation and functionality for further mechanistic insights.

## Supplementary Materials

The following are available online at https://www.mdpi.com/2076-393X/8/2/271/s1. Figure S1: HOCl induces significant changes in protein expression in melanoma cancer cells revealed by proteomic analysis of A375 melanoma cells. (a) Heat map of Pearson coefficient correlation analysis among tested conditions. (b) Principal component analysis. (c) Volcano plot representation of t test analysis comparing protein intensities in Ox-L versus FT-L; each point represent one protein. Proteins located above the lines are statistically significantly modulated in their expression level (FDR = 0.05, S0 = 1), Figure S2: Oxidized peptide occurrence is not increase in DCs after loading with HOCl oxidized tumor lysate. (**a**) Volcano plot representation of t test analysis comparing peptide intensities in immature DCs loaded with Ox-L versus FT-L; each point represent one peptide plotted by log2 fold change versus minus logarithm of the *q*-value (Benjamini-Hochberg corrected *p*-value), with a cutoff value of 0.05; in red peptides presenting at least one oxidized site. (**b**) Same as (a) for mature DCs, Figure S3: Treating the antigen source with HOCl does not significantly impact on the protein levels of antigen processing machinery components in mo-DCs. Heat map and relative sample clustering of Z score of Log2 intensities (LFQ values) of selected proteins (gene names indicated on the left column) known to be involved in antigen processing and presentation [50,51]. Sample abbreviations: D: donor; im: immature mo-DCs; m: mature mo-DCs; R: technical replicate, Figure S4: HOCl incubation does not completely disrupt HLA-peptide complexes in tumor cells. A375 melanoma tumor cells were incubated for 1 h in the presence (Ox-L) or absence (FT-L) of 60 μM HOCl, washed and subjected to 6x freeze-thaw cycles, or left untouched after harvesting (pellet). HLA-I and –II binding peptides were extracted and identified by MS. (**a**,**c**) Venn diagram reporting the number of peptides isolated from HLA-I (a) or HLA-II (**d**) complexes between the indicated conditions (blue: pellet; orange: FT-L; grey: Ox-L). (b,d) Average numbers of peptides isolated from HLA-I (**b**) or HLA-II (d) complexes in the indicated conditions with standard deviation (*n* = 3 biological replicates, each analyzed in duplicate). (**e**) Clusters of recurrent binding motifs for HLA-I isolated peptides, calculating using online source: http://www.cbs.dtu.dk/services/GibbsCluster/. Data were analyzed with unpaired student *t*-test: * *p* < 0.05; *** *p* < 0.001, Figure S5: Peptide length distribution is equivalent among peptides with source protein reported 3D structures in the PDB database, compared to all the peptides experimentally determined (with or without 3D for the source protein) for both Ox-L and FT-L HLA-II ligandomes. Graph reporting the relative abundance in % (y-axis) of peptides with the reported amino acid length (x-axis) in the indicated peptide collections. Legend: HLA-II-FT-L: HLA-II ligand peptides identified in mo-DCs loaded with FT-L, HLA-II-FT-L-PDB: a subset of HLA-II-FT-L with reported 3D structures for their respective source proteins, HLA-II-Ox-L: HLA-II ligand peptides identified in mo-DCs loaded with Ox-L, HLA-II-Ox-L-PDB: a subset of HLA-II-Ox-L with reported 3D structures for their respective source proteins, Figure S6: Amino acid composition is equivalent among peptides with source protein reported 3D structures in the PDB database, compared to all the peptides experimentally determined (with or without 3D structures for the source protein), for both Ox-L and FT-L HLA-II ligandome. Graph reporting the amino acid frequencies for the indicated peptide collections. (Legend details same as Supplemental Figure S5). Amino acid frequencies were obtained by computing the number of occurrences of a given amino acid divided by the total number of amino acids in the respective condition, Table S1: HLA typing of the A375 melanoma cell line (retrieved from [74]) and of donor 1 cells used for immunopeptidomics analysis, Table S2: List of peptides and their respective PTMs identified in the A375 proteomics dataset. MaxQuant output modificationSpecificPeptides.txt table was filtered to remove reverse and potential contaminants, Table S3: List of proteins and their respective PTMs identified in the A375 proteomics dataset. MaxQuant output proteinGroups.txt table was filtered to remove reverse and potential contaminants and protein intensities were log2 transformed, Table S4, List of proteins and their relative PTMs identified in the mo-DC proteomics dataset generated in this work. MaxQuant output proteinGroups.txt table was filtered to remove reverse and potential contaminants and protein intensities were log2 transformed, Table S5: List of HLA-I and HLA-II peptides identified in the A375 immunopeptidomics dataset. MaxQuant output peptides.txt table was filtered to remove reverse and potential contaminants and peptide intensities were log2 transformed, Table S6: List of HLA-I and HLA-II peptides identified in the mo-DC immunopeptidomics dataset. MaxQuant output peptides.txt table was filtered to remove reverse and potential contaminants and peptide intensities were log2 transformed.
