# Supplementary material for: Deciphering the Mechanisms of Improved Immunogenicity of Hypochlorous Acid-Treated Antigens in Anti-Cancer Dendritic Cell-Based Vaccines
Source: Vaccines (Basel). 2020 Jun 2;8(2):271. doi: 10.3390/vaccines8020271 (PMC7349990; doi:10.3390/vaccines8020271)
Supplement: Supplementary file 1 [file vaccines-08-00271-s001.zip › supplementary table 1.pdf]

	HLA-I			HLA-II		
A375 cell line	A*01:01<br>A*02:01	B*57:01<br>B*44:03	C*16:02<br>C*06:02	DQA1*03:02<br>DQA1*02:01	DQB1*03:03<br>DQB1*03:03	DRB*:04:05<br>DRB1*07:01
Donor 1	A*03:01<br>A*24:02	B*07:02<br>B*35:03	C*07:02<br>C*12:03	DQA1*01:04<br>DQA1*05:05	DQB*:03:01<br>DQB1*05:03	DRB1*12:01<br>DRB1*14:54

Supplemental Table 1. HLA typing of the A375 melanoma cell line (retrieved from (1)) and of donor 1 cells used for immunopeptidomics analysis.

References:

1. Boegel, S.; Löwer, M.; Bukur, T.; Sahin, U.; Castle, J.C. A catalog of HLA type, HLA expression, and neoepitope candidates in human cancer cell lines. *Oncoimmunology* **2014**, *3*, e954893, doi:10.4161/21624011.2014.954893.
