# Supplementary material for: Deciphering the Mechanisms of Improved Immunogenicity of Hypochlorous Acid-Treated Antigens in Anti-Cancer Dendritic Cell-Based Vaccines
Source: Vaccines (Basel). 2020 Jun 2;8(2):271. doi: 10.3390/vaccines8020271 (PMC7349990; doi:10.3390/vaccines8020271)
Supplement: Supplementary file 1 [file vaccines-08-00271-s001.zip › Supplementary Figures.pdf]

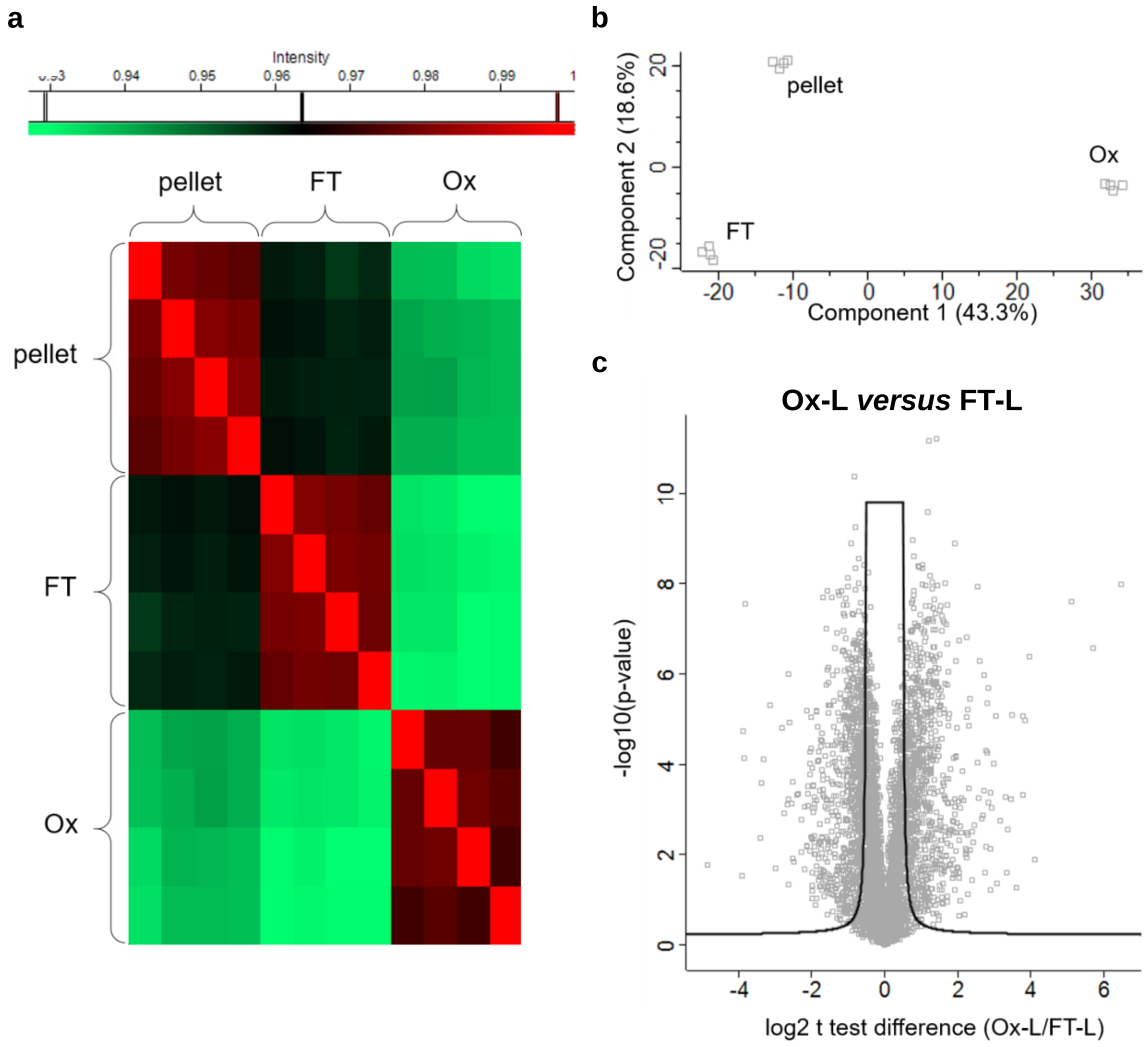

Supplemental Figure 1. HOCl induces significant changes in protein expression in melanoma cancer cells revealed by proteomic analysis of A375 melanoma cells. (**a**) Heat map of Pearson coefficient correlation analysis among tested conditions. (**b**) Principal component analysis. (**c**) Volcano plot representation of t test analysis comparing protein intensities in Ox-L *versus* FT-L; each point represent one protein. Proteins located above the lines are statistically significantly modulated in their expression level (FDR = 0.05, S0 = 1).

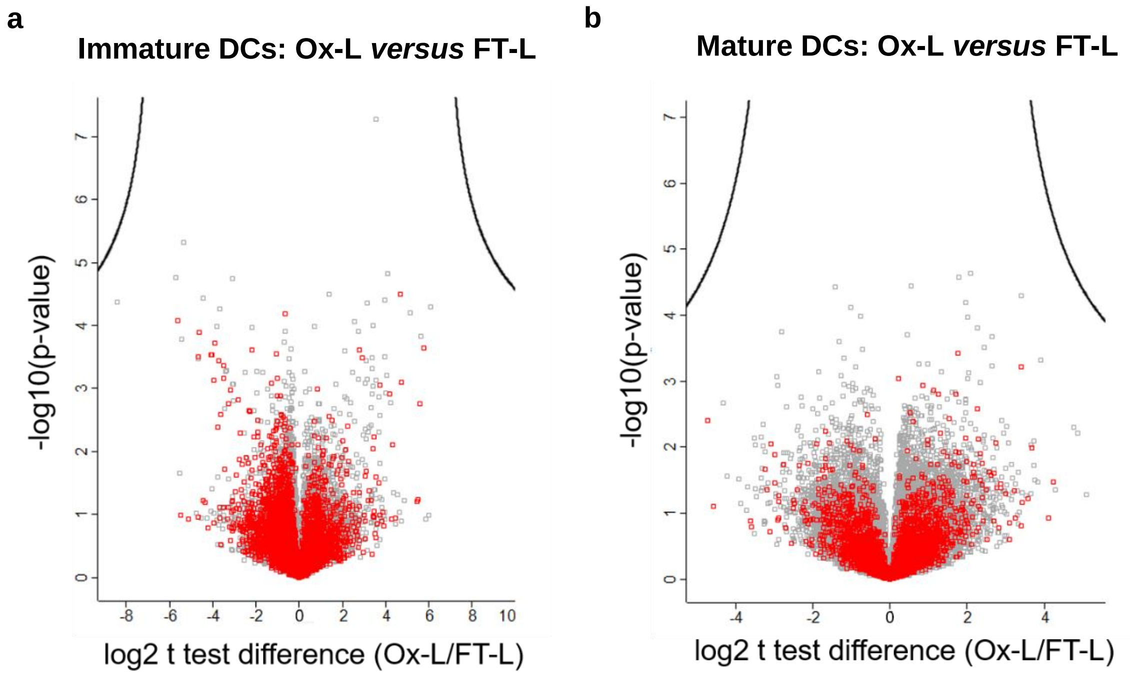

Supplemental Figure 2. Oxidized peptide occurrence is not increase in DCs after loading with HOCl oxidized tumor lysate. (**a**) Volcano plot representation of t test analysis comparing peptide intensities in immature DCs loaded with Ox-L *versus* FT-L; each point represent one peptide plotted by log2 fold change versus minus logarithm of the q-value (Benjamini-Hochberg corrected p-value), with a cutoff value of 0.05; in red peptides presenting at least one oxidized site. (**b**) Same as (**a**) for mature DCs.

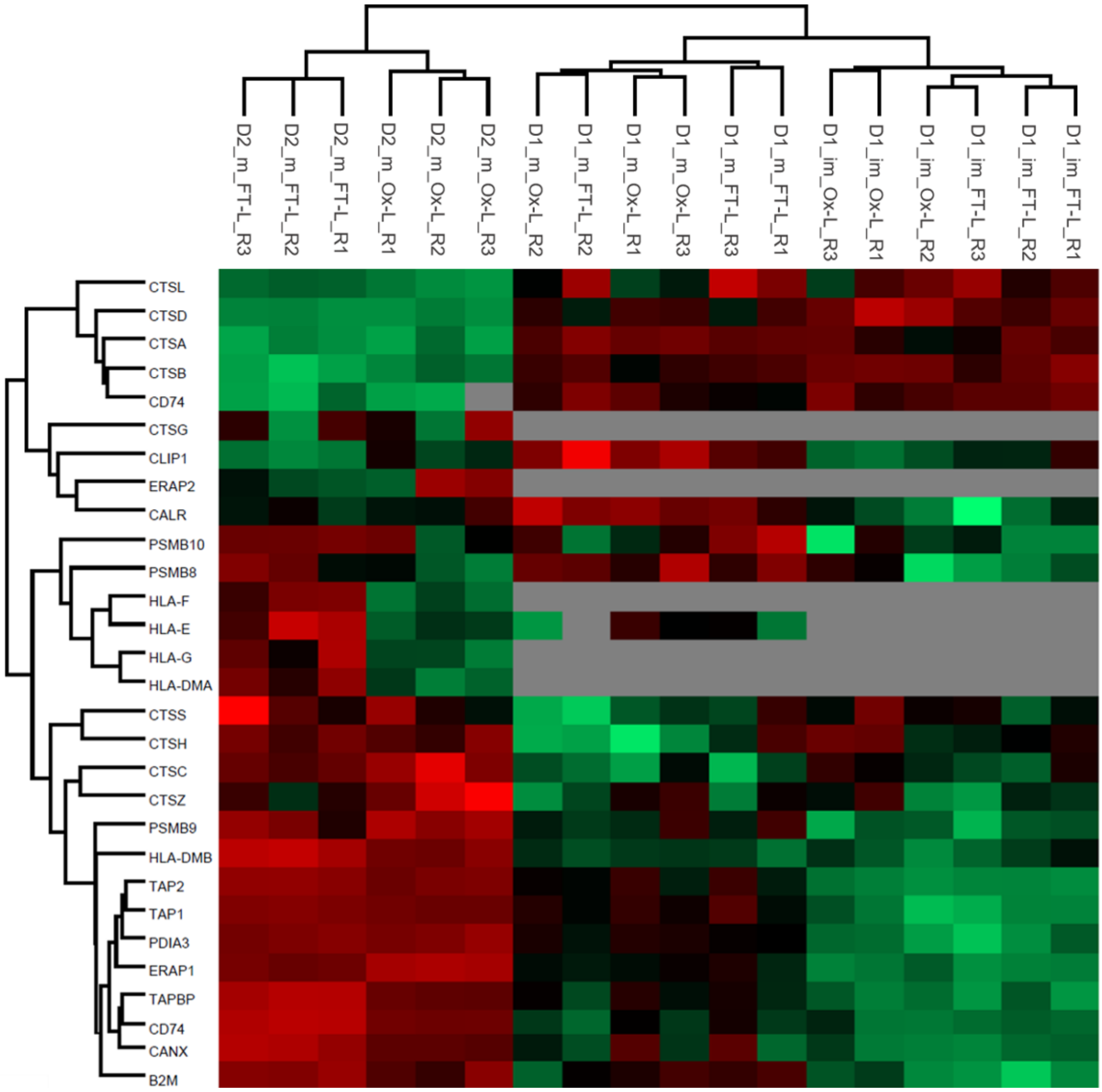

Supplemental Figure 3. Treating the antigen source with HOCl does not significantly impact on the protein levels of antigen processing machinery components in mo-DCs. Heat map and relative sample clustering of Z score of Log2 intensities (LFQ values) of selected proteins (gene names indicated on the left column) known to be involved in antigen processing and presentation [50,51]. Sample abbreviations: D: donor; im: immature mo-DCs; m: mature mo-DCs; R: technical replicate.

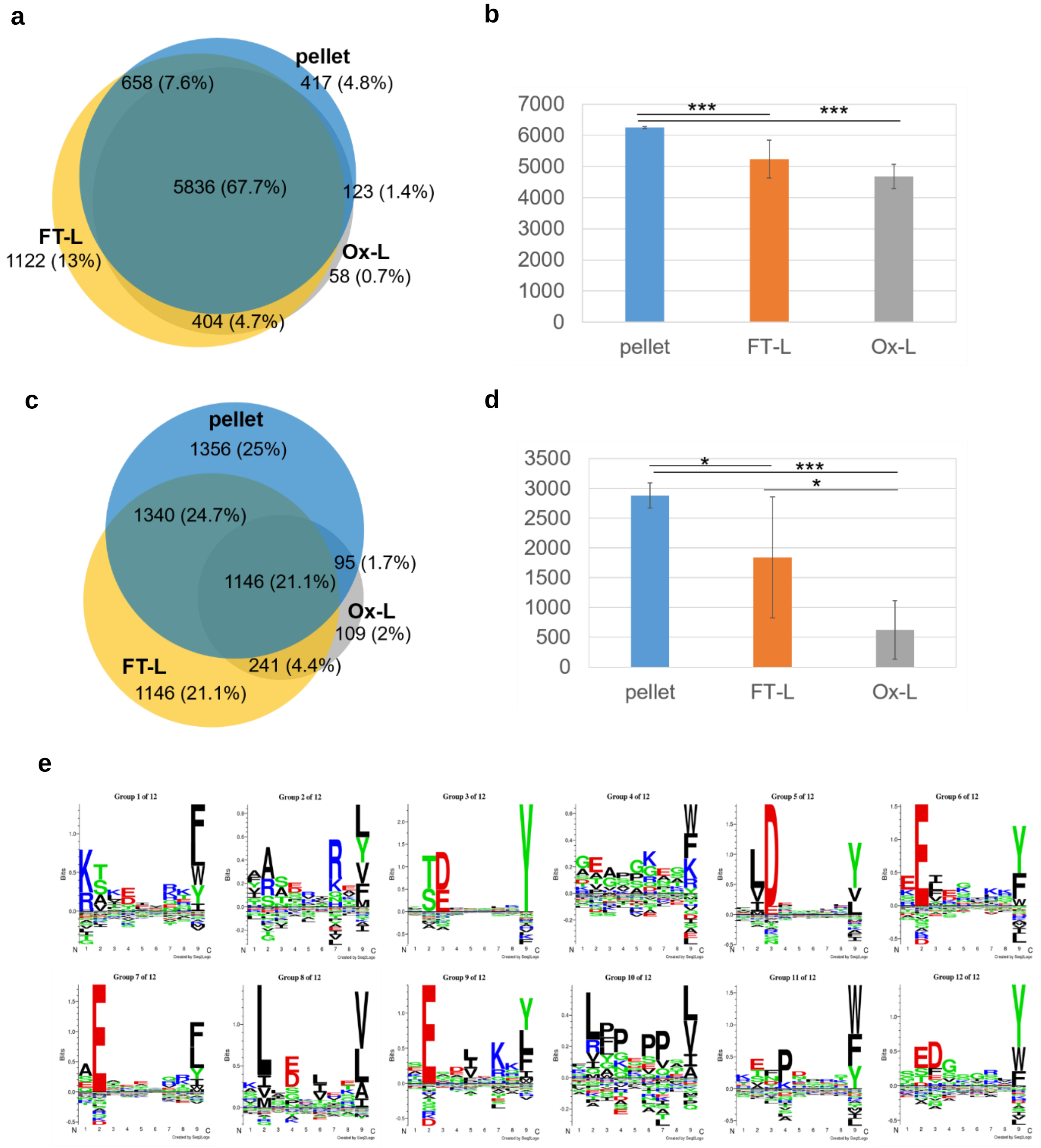

Supplemental Figure ~~3~~4. HOCl incubation do not completely disrupt HLA-peptide complexes in tumor cells. A375 melanoma tumor cells were incubated for 1hr in the presence (Ox-L) or absence (FT-L) of 60µM HOCl, washed and subjected to 6x freeze-thaw cycles, or left untouched after harvesting (pellet). HLA-I and –II binding peptides were extracted and identified by MS. (**a**) Venn diagram reporting the number of peptides isolated from HLA-I complexes between the indicated conditions (blue: pellet; orange: FT-L; grey: Ox-L). (**b**) Average numbers of peptides isolated from HLA-I complexes in the indicated conditions with standard deviation (n=3 biological replicates, each analyzed in duplicate). (**c**) Venn diagram reporting the number of peptides isolated from HLA-II complexes between the indicated conditions (blue: pellet; orange: FT-L; grey: Ox-L). (**d**) Average numbers of peptides isolated from HLA-II complexes in the indicated conditions with standard deviation (n=3 biological replicates, each analyzed in duplicate). (**e**) Clusters of recurrent binding motifs for HLA-I.

isolated peptides, calculating using online source: http://www.cbs.dtu.dk/services/GibbsCluster/. Data were analyzed with unpaired student t-test: *:$p < 0.05$; ***:$p < 0.001$.

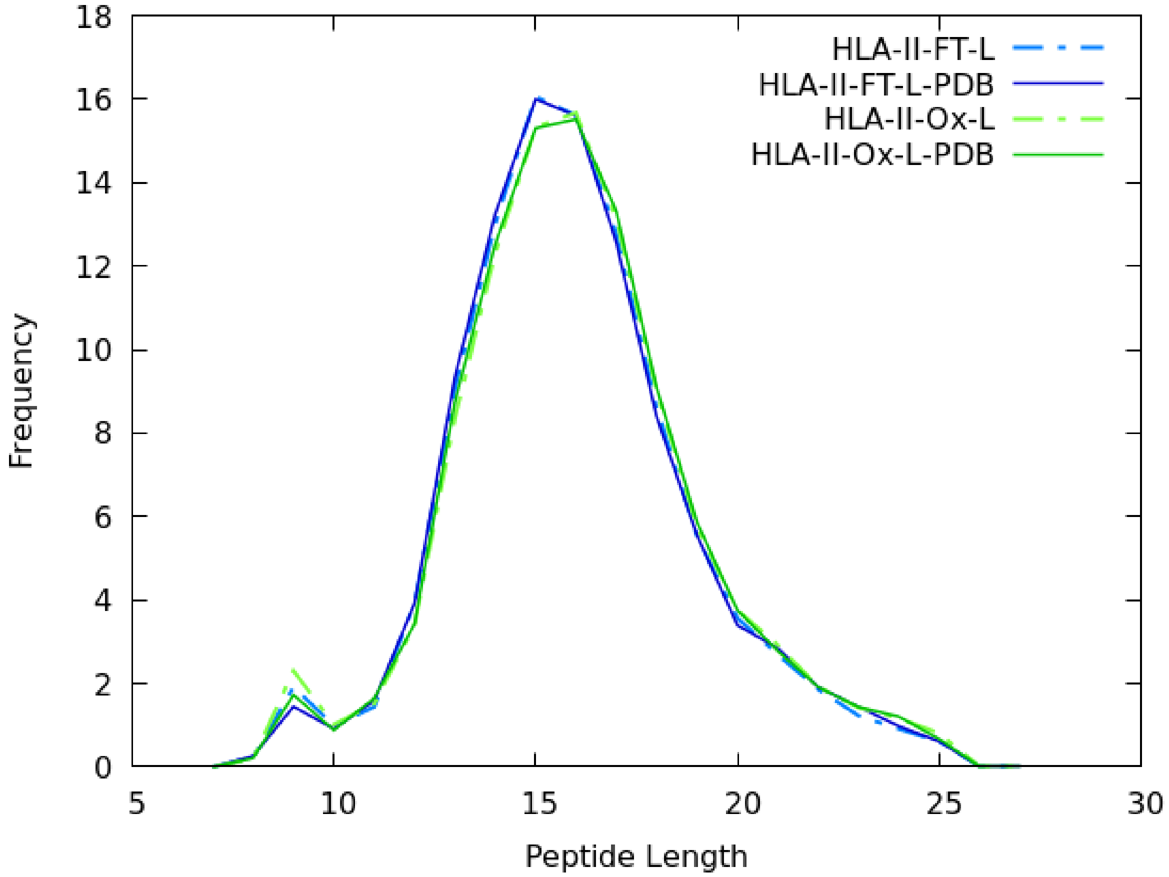

Supplemental Figure 54. Peptide length distribution is equivalent among peptides with source protein reported 3D structures in the PDB database, compared to all the peptides experimentally determined (with or without 3D for the source protein) for both Ox-L and FT-L HLA-II ligandomes. Graph reporting the relative abundance in % (y-axis) of peptides with the reported amino acid length (x-axis) in the indicated peptide collections. Legend: HLA-II-FT-L: HLA-II ligand peptides identified in mo-DCs loaded with FT-L, HLA-II-FT-L-PDB: a subset of HLA-II-FT-L with reported 3D structures for their respective source proteins, HLA-II-Ox-L: HLA-II ligand peptides identified in mo-DCs loaded with Ox-L, HLA-II-Ox-L-PDB: a subset of HLA-II-Ox-L with reported 3D structures for their respective source proteins. Equivalent peptide length distributions were observed between the original set and subset with PDB representation, indicating that the subsets with PDB representation are representative of the set experimentally determined.

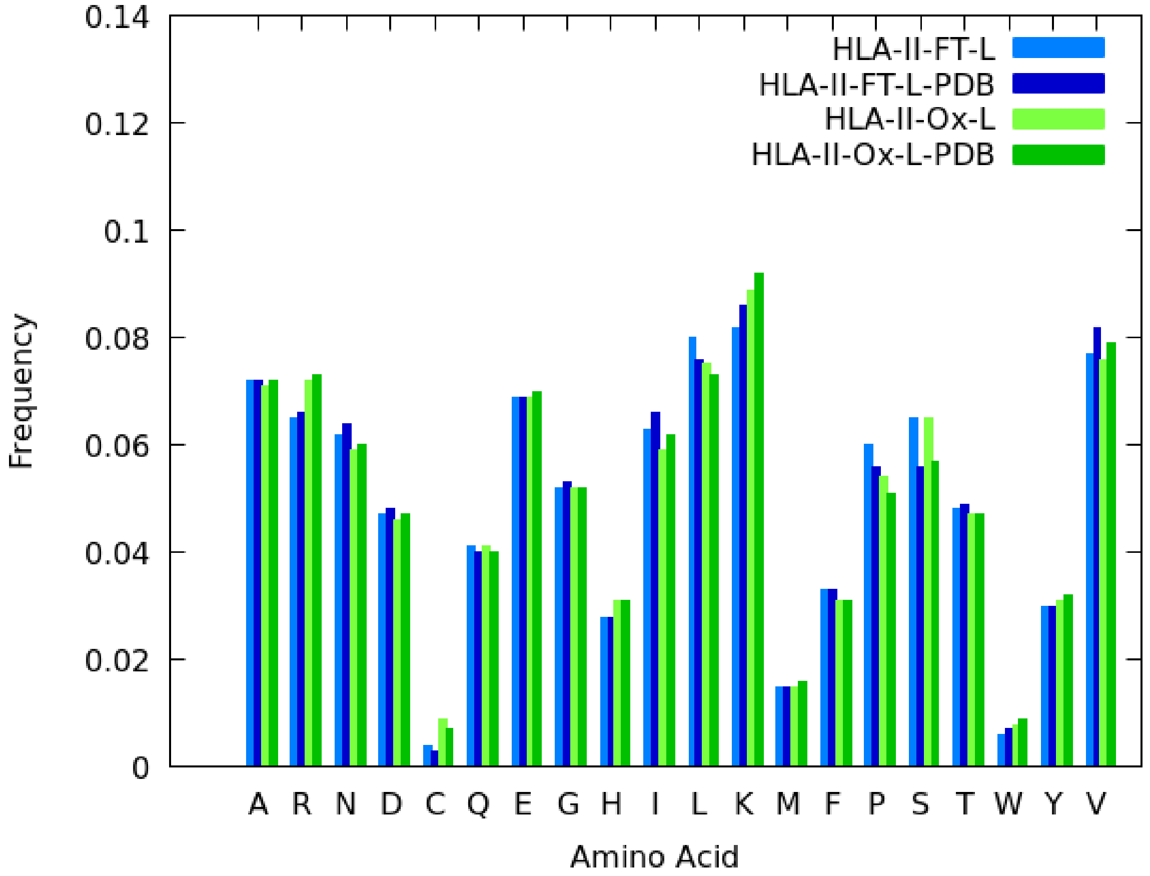

Supplemental Figure 6~~5~~. Amino acid composition is equivalent among peptides with source protein reported 3D structures in the PDB database, compared to all the peptides experimentally determined (with or without 3D structures for the source protein), for both Ox-L and FT-L HLA-II ligandome. Graph reporting the amino acid frequencies for the indicated peptide collections. (Legend details same as **Supplemental Figure 54**). Amino acid frequencies were obtained by computing the number of occurrences of a given amino acid divided by the total number of amino acids in the respective condition. Equivalent amino acid distributions are observed between the original set and subset with PDB representation, indicating that the subsets with PDB representation are representative of the set experimentally determined. One single exception was observed for the amino acid Serine whose occurrence resulted 0.8% lower in the subsets with PDB representation for both FT-L and OX-L conditions. This effect is negligible when comparing solvent accessibilities between FT-L and OX-L (as the same decrease is observed in FT-L and in Ox-L conditions).
